# The Interactions in the Carboxyl Terminus of Human 4-Hydroxyphenylpyruvate Dioxygenase Are Critical to Mediate the Conformation of the Final Helix and the Tail to Shield the Active Site for Catalysis

**DOI:** 10.1371/journal.pone.0069733

**Published:** 2013-08-09

**Authors:** Jang-Foung Lin, Yung-Lin Sheih, Tsu-Chung Chang, Ni-Yuan Chang, Chiung-Wen Chang, Chia-Pei Shen, Hwei-Jen Lee

**Affiliations:** Department of Biochemistry, National Defense Medical Center, Neihu, Taipei, Taiwan; University of Edinburgh, United Kingdom

## Abstract

4-Hydroxylphenylpyruvate dioxygenase (4-HPPD) is an important enzyme for tyrosine catabolism, which catalyzes the conversion of 4-hydroxylphenylpyruvate (4-HPP) to homogentisate. In the present study, human 4-HPPD was cloned and expressed in *E. coli*. The kinetic parameters for 4-HPP conversion were: *k*_cat_ = 2.2±0.1 s^−1^; and *K*_m_ = 0.08±0.02 mM. Sequence alignments show that human 4-HPPD possesses an extended C-terminus compared to other 4-HPPD enzymes. Successive truncation of the disordered tail which follows the final α-helix resulted in no changes in the *K*_m_ value for 4-HPP substrate but the *k*_cat_ values were significantly reduced. The results suggest that this disordered C-terminal tail plays an important role in catalysis. For inspection the effect of terminal truncation on protein structure, mutant models were built. These models suggest that the different conformation of E254, R378 and Q375 in the final helix might be the cause of the activity loss. In the structure E254 interacts with R378, the end residue in the final helix; mutation of either one of these residues causes a *ca.* 95% reductions in *k*_cat_ values. Q375 provides bifurcate interactions to fix the tail and the final helix in position. The model of the Q375N mutant shows that a solvent accessible channel opens to the putative substrate binding site, suggesting this is responsible for the complete loss of activity. These results highlight the critical role of Q375 in orientating the tail and ensuring the conformation of the terminal α-helix to maintain the integrity of the active site for catalysis.

## Introduction

**Figure 1:**
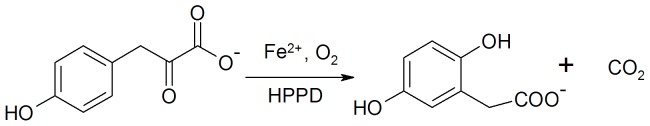
Catalytic conversion of HPP to HGA by HPPD.

4-Hydroxylphenylpyruvate dioxygenase (4-HPPD, EC 1.13.11.27) belongs to the non-haem Fe(II)/2-oxoacid-dependent oxygenase superfamily, which couples the oxidative decarboxylation of a 2-oxoacid (most commonly α-ketoglutarate) to the oxidation of the prime substrate. A wide range of different types of reactions are catalyzed by these oxygenases, including hydroxylations, desaturations and oxidative ring closures. These reactions have environmental, pharmaceutical and medical significance [1], [2]. 4-HPPD catalyzes the second step in the pathway of tyrosine catabolism, the conversion of 4-hydroxyphenyl-pyruvate (4-HPP) to homogentisate (HG) (Fig. 1). The conversion of substrate involves oxidative decarboxylation, side-chain migration and aromatic hydroxylation in a single catalytic cycle [3]. The 4-HPPD reaction is unusual in that the α-ketoacid and prime substrate moieties are contained within the same molecule. A deficiency in active 4-HPPD in humans results in type III tyrosinemia, a rare autosomal recessive disorder [4]. In plants the homogensate reaction product is an intermediate in the biosynthesis of plastoquinone and tocopherols [5], and inhibitors of 4-HPPD have been used as herbicides [6]–[8].

Human 4-HPPD is active as a homodimer with a subunit molecular mass of *ca.* 45 kDa [9]. Alignment of the amino acid sequences of 4-HPPD from different species shows that they are less than 30% identical. However, the topologies of all determined 4-HPPD structures are very similar. The structure of 4-HPPD is comprised of two barrel-shaped domains and is similar in topology to the extradiol dioxygenases [10]–[16]. A 2-His-1-carboxylate motif is buried inside the β–barrel of the carboxyl-terminal domain of 4-HPPD which binds the iron(II) cofactor [10]–[13], [16]. This metal binding motif is strictly conserved among non-haem iron(II)-dependent oxygenases [17], [18].

**Figure 2:**
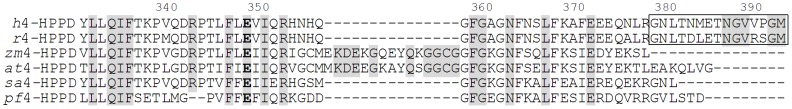
Alignment of amino acid sequences of the C-terminus of human 4-HPPD with enzymes from other species [37]. Fully conservative sequences and residues in iron binding sphere are colored grey and dark grey, respectively. Abbreviations used: *h*4-HPPD, *Homo sapiens* (human) 4-HPPD; *r*4-HPPD, *Rattus norvegicus* (rat) 4-HPPD; *zm*4-HPPD, *Zea mays* 4-HPPD; *at*4-HPPD, *Arabidopsis thaliana* 4-HPPD; *sa*4-HPPD, *Streptomyces avermitilis* 4-HPPD; *pf*4-HPPD, *Pseudomonas fluorescens* 4-HPPD. The sequences for the *h*4-HPPD and *r*4-HPPD C-terminal tail (G379 to M393) are highlighted by the square frame.

The active site of 4-HPPD is buried inside a barrel-like β-sheet which is shielded with a C-terminal α-helix [10]–[13], [16]. Covering of the active site by a C-terminal extension is commonly observed in many 2-oxoglutarate-dependent oxygenases and it is assumed that the C-terminus functions as a gate and controls access to the active site and isolates the bound substrate during catalysis [11], [19]–[22]. Superimposing the crystal structures of *P. fluorescens* 4-HPPD and *S. avermitilis* 4-HPPD in complex with NTBC reveal significant differences in the position of C-terminal helix [10], [12]. Binding of the NTBC inhibitor in the active site leads to a 40 degree rotation of the C-terminal α-helix. Residues in the terminal α-helix might also be involved in catalysis [23], [24]. For example, replacement of F337 and F341, two residues in the terminal α-helix in *S. avermitilis* 4-HPPD, by Ile and Tyr resulted in loss of activity [23]. The aromatic side-chain of F337 is thought to interact with the aromatic ring of the substrate by π-π interactions [23], [25]. Human and rat 4-HPPD possess longer C-terminal sequences than enzymes from plants and microorganisms (Fig. 2). Truncation experiments suggest that the C-terminal extension is essential for enzyme activity [26], but little is known about its function as the residues beyond the final C-terminal α-helix are disordered in all reported X-ray crystal structures [10]–[13], [16]. To date, the precise role of the C-terminus has not been determined.

This study reports the effect of truncating successive C-terminal residues on the activity of recombinant human 4-HPPD. Activity is progressively reduced upon truncation of the C-terminus, indicating the important role of this tail in catalysis. Structural modeling of truncation mutants was carried out using the X-ray coordinates of human 4-HPPD to investigate the effects of C-terminal truncation on protein structure [16]. All models were geometrically minimized by the quantum mechanical-molecular mechanical calculations. The truncated mutant models showed a different conformation in the terminal helix, especially a change in conformation of the benzene ring of F371 and large differences in the side-chain conformations of E254, R378 and Q375. In the structure, the residues of R378 and Q375, which are located in the final helix, provide interactions which fix this helix and the C-terminal tail into position. Substitution of Q375 and R378 resulted in loss of activity, indicating that these interactions are critical for maintaining the helix in a stable conformation for catalysis. The Q375N mutant model showed a solvent accessible channel opened from the putative substrate binding site, suggesting the interactions provided by Q375 to hold the terminal helix and the tail in proper position are critical for isolating the active site from solvent during catalysis.

## Materials and Methods

### Materials

The restriction enzymes and T4 DNA ligase used for cloning were purchased from NEBioLab (Beverly, MA) and the QuikChange site-directed mutagenesis kit from Stratagene (La Jolla, CA). HiPrep 16/10 Q XL and Sephacryl HR-100 columns were purchased from GE Healthcare (Fairfield, CT). Q-Sepharose, SOURCE 15PHE and Sephacryl HR-100 were purchased from GE Healthcare (Uppsala, Sweden). All other chemicals and buffers were obtained from the Sigma-Aldrich Chemical Co. (St. Louis, MO) or J. T. Baker (Phillipsburg, NJ) and were of the highest purity available.

### Cloning

HepG2 cells were grown in Dulbecco's modified Eagle medium for 4 days to 5×10^6^ cells before harvesting and homogenized in buffer RX for total RNA preparation (Viogen Total RNA Miniprep System). The first strand of cDNA was obtained by reverse transcription using the BRL ThermoScript™ RT-PCR system and an oligo(dT) primer according to the instructions of the manufacturer. The 4-HPPD gene was amplified by PCR using the following primers: forward primer 5′-GGAATTCCATATGATGACGACTTACAGTGACAAAGGGGCA-3′ and reverse primer 5′-CGGGGATCCCTACATGCCGGGCACCACCCCATTGGT-3′. These primers were designed based on the sequence of human liver 4-HPPD (accession no: P32754), with additional *Nde*I and *BamH*I restriction sites. The amplification reaction mixture contained 1 µL cDNA, 0.4 mM dNTPs, 0.2 µM forward and reverse primers, and 1 unit Taq DNA polymerase (Qiagen). After initial denaturation at 95°C for 5 min, amplification was performed by 30 cycles of the following: 95°C for 30 s; 58°C for 30 s; and 72°C for 2 min; followed by a final extension at 72°C for 10 min. The purified PCR product after digestion by *Nde*I and *BamH*I restriction enzymes was cloned into the pTrc vector. The integrity of the plasmid with the 4-HPPD gene (termed as p*Trc*-4-HPPD) was confirmed by DNA sequencing.

### Preparation of mutants

Truncated mutants of 4-HPPD were prepared by PCR using p*Trc*-4-HPPD as template. The forward and reverse primers used in PCR are shown in Table 1. Preparation of point mutants was carried out using the QuikChange mutagenesis system. Two complementary primers including the desired mutations (Table 1), template vector (p*Trc*-4-HPPD) and *PfuTurbo* DNA polymerase were used for PCR. After amplification, parental DNA was digested with *Dpn*I and newly synthesized mutant-containing vectors were transformed into *E. coli* DH5α competent cells. To confirm the presence of the desired mutation, the complete DNA sequences of the mutant 4-HPPD enzymes were determined.

**Table 1:** Primer sequences used to perform mutagenesis experiments.

Mutation site	Primer sequences
	For C-terminal truncation mutants*
	Forward primer:
	5′-GTCAAGGGTGGTCATATGACGACTTACAGTGACAAAGGGGCA
	Reverse primers:
ΔG388	5′-CGGGGATCCCTACCCATTGGTCTCCATGTTGGTGAG
ΔE385	5′-CGGATTGGATCCTCACTCCATGTTGGTGAGGTTACC
ΔL381	5′-GAACGGATTGGATCCTCAGAGGTTACCCCGCAGGTTCTGCTC
ΔN380	5′-CTGATTGGATCCTCAGTTACCCCGCAGGTTCTGCTC
ΔG379	5′-CGGGGATCCCTAACCCCGCAGGTTCTGCTCCTCCTC
ΔR378	5′-CGGGGATCCCTACCGCAGGTTCTGCTCCTCCTCGAA
	For point mutants†
R378K	5′-GAGGAGCAGAACCTGAAGGGTAACCTCACC
Q375N	5′-TTCGAGGAGGAGAACAACCTGCGGGGTAAC
R378K/Q375N	5′-GAGGAGGAGAACAACCTGAAGGGTAACCTC
E254D	5′-GGCAAGAAGAAGTCCCAGATCCAGGACTATGTGG
	(and the respective complementary primers)

* The restriction enzyme cutting sites are underlined.

† The mutation sites are underlined.

### Protein expression and purification

Expression of wild-type and mutant 4-HPPD was similar to previously reported methods with slight modification [26]. Clones of wild-type or mutant p*Trc*-4-HPPD genes were fermented at 27°C in 2YT broth supplemented with 30 µg/mL chloramphenicol for 26 hrs. Cells were harvested by centrifugation and kept frozen at −80°C until use.

Cell pellets were resuspended and sonicated in buffer A (containing 50 mM Tris-HCl buffer, pH 7.5, 1 mM EDTA, and 1 mM DTT). After centrifugation at 12,000 g for 15 min at 4°C, the supernatants were loaded onto Q-Sepharose anion exchange column (26 mm×15 cm) equilibrated in the same buffer. Recombinant 4-HPPD was eluted during the washing step. Fractions were pooled based on the presence of a 45 kDa band upon SDS-PAGE analyses and ammonium sulfate solution was added to a final concentration of 1.2 M. The sample was filtered and loaded onto SOURCE 15PHE column (16×100 mm) equilibrated with buffer A supplemented with 1.2 M (NH_4_)_2_SO_4_ and 10% (v/v) glycerol and eluted with a gradient into buffer A supplemented with 10% (v/v) glycerol over 100 mL with fractions of 2 mL collected. Recombinant 4-HPPD was eluted at approximately 0.6 M (NH_4_)_2_SO_4_. Fractions were pooled, concentrated to 8 mL, and loaded onto S-100 Sephacryl column (26×900 mm) equilibrated with 50 mM Tris-HCl buffer, pH 7.5. Fractions exhibited the highest purity were pooled and concentrated.

### Western blot analysis

After separation of wild-type and mutant 4-HPPD by 10% (w/v) SDS-PAGE, the proteins were electrophoretically transferred onto PVDF membrane by Semi-dry transfer (BioRad, USA). Membranes were incubated for 90 min in PBST buffer (10 mM Tris-HCl, pH 7.6, 150 mM NaCl and 0.1% (v/v) Tween 20) containing 5% (v/v) defatted milk. The membrane was treated with anti-rabbit-4-HPPD antibody (Bioman Scientific Co., Taiwan) in PBST buffer for 1 h, followed by goat anti-rabbit IgG horseradish peroxidase conjugate (Santa Cruz, CA) for 1 h. Reactions were detected by peroxidase activity and enhanced chemiluminescence (ECL, GE Healthcare) following the manufacture's instruction.

### Enzyme activity measurements

#### 1. Oxygen consumption by Oxygraph

The activity of 4-HPPD was measured using oxygen consumption in a Hansatech DW1 Oxygraph System (Hansatech, UK) equipped with a Clark-type electrode. The reaction mixture (total 2 mL containing 0.2 mM ascorbate, 0.2 mM ferrous sulfate and 5 µg 4-HPPD in 0.1 M Tris-acetate buffer, pH 6.5) was incubated for 1 min at 37°C, before addition of 1 mM 4-HPP to initiate the reaction. Reaction rates were corrected for consumption of oxygen in the absence of 4-HPPD. The concentrations of 4-HPP used in kinetic assays varied between 0.01 to 2 mM. The apparent kinetic parameters were determined by fitting to the Michaelis-Menten equation using SigmaPlot software (Systat Software).

#### 2. Assay of enzyme activity by HPLC

Reaction mixtures (200 µL) containing 0.1 M Tris-acetate, pH 6.5, 4 mM ascorbate, 1 mM ferrous sulfate, 1 mM 4-HPP and 5 µg 4-HPPD were incubated for 5 min at 37°C. After incubation, the reaction mixtures were filtered by centrifugation at 8000 g (an ultrafiltration spin column from Vivascience with molecular weight cut of 5 kDa) to remove the enzyme. The products were analysed by HPLC on a C18 column (4.6×250 mm, 5-µm particle size; ODS HYPERSIL) at a flow rate of 1 mL/min. Two solvents [0.1% (v/v) TFA for buffer A; and 0.07% (v/v) TFA/80% (v/v) acetonitile for buffer B] were used with the following gradient: 17 min linear gradient from 100% buffer A to 30% buffer A/70% buffer B; 2 min linear gradient to 100% buffer B; and 1 min 100% buffer B. The elution was monitored at 288 or 230 nm.

### Circular dichroism studies

Circular dichroism (CD) spectra were measured using a Jasco J-815 spectropolarimeter equipped with a Peltier temperature controller accessory. Experiments were performed using 1 mm path length cell for the far-UV region (200 to 250 nm). All spectra were averaged from three accumulations and were buffer corrected.

### Building models of human 4-HPPD

Modeling of human 4-HPPD in complex with 4-HPP was generated using the Discovery Studio 2.5 software (Accelrys Inc.). Structures of human 4-HPPD (PDB code: 3ISQ) [16] were used as the starting model. The 4-HPP substrate was docked into the active site of *h*4-HPPD using the LibDock protocol. One hundred hotspots in the binding pocket of protein were set for conformer matching. The binding pocket was defined as a 10 Å radius sphere centred at the iron(II) atom for calculation and smart minimization. The high quality mode and best conformation method were selected for docking. Other parameters were used at their default settings. After docking, a series of 4-HPP conformations were applied to energy minimization with the algorithm of smart minimization until the gradient tolerance was satisfied (RMS Gradient ∼0.1 kcal/mol/Å).

Mutant 4-HPPD was built using the Built Mutants protocol followed by energy minimization. Quantum mechanical-molecular mechanical (QM-MM) calculations were performed on the models of wild-type and mutant enzymes in complex with 4-HPP using the Minimization (QM-MM) protocol. Before the simulation, atom constraints were applied to the regions outside 5 Å distance of 4-HPP and the two residues of Q375 and R378. Inside the regions were simulated and the metal ion, 4-HPP and the side-chains of H183, H266, E349, Q334, Q265 and Q251 were defined as the quantum atoms. The parameter of Multiplicity in DMol3 was set as auto. All other parameters of the protocol were used with their default setting.

## Results

### Purification of recombinant human 4-HPPD

Recombinant human liver 4-HPPD gene was cloned from HepG2 cells. The sequence of the cloned 4-HPPD gene was identical to that reported for human liver enzyme (EMBL sequence data bank access number: X72389) [27]. When *E. coli* cells harbouring recombinant 4-HPPD gene were cultured a characteristic brownish pigment was observed, as reported previously [27], [28]. This pigment is an oxidation product of homogentisate [27], [29]. This observation indicates soluble expression of active enzyme from the recombinant gene in *E. coli*.

**Figure 3:**
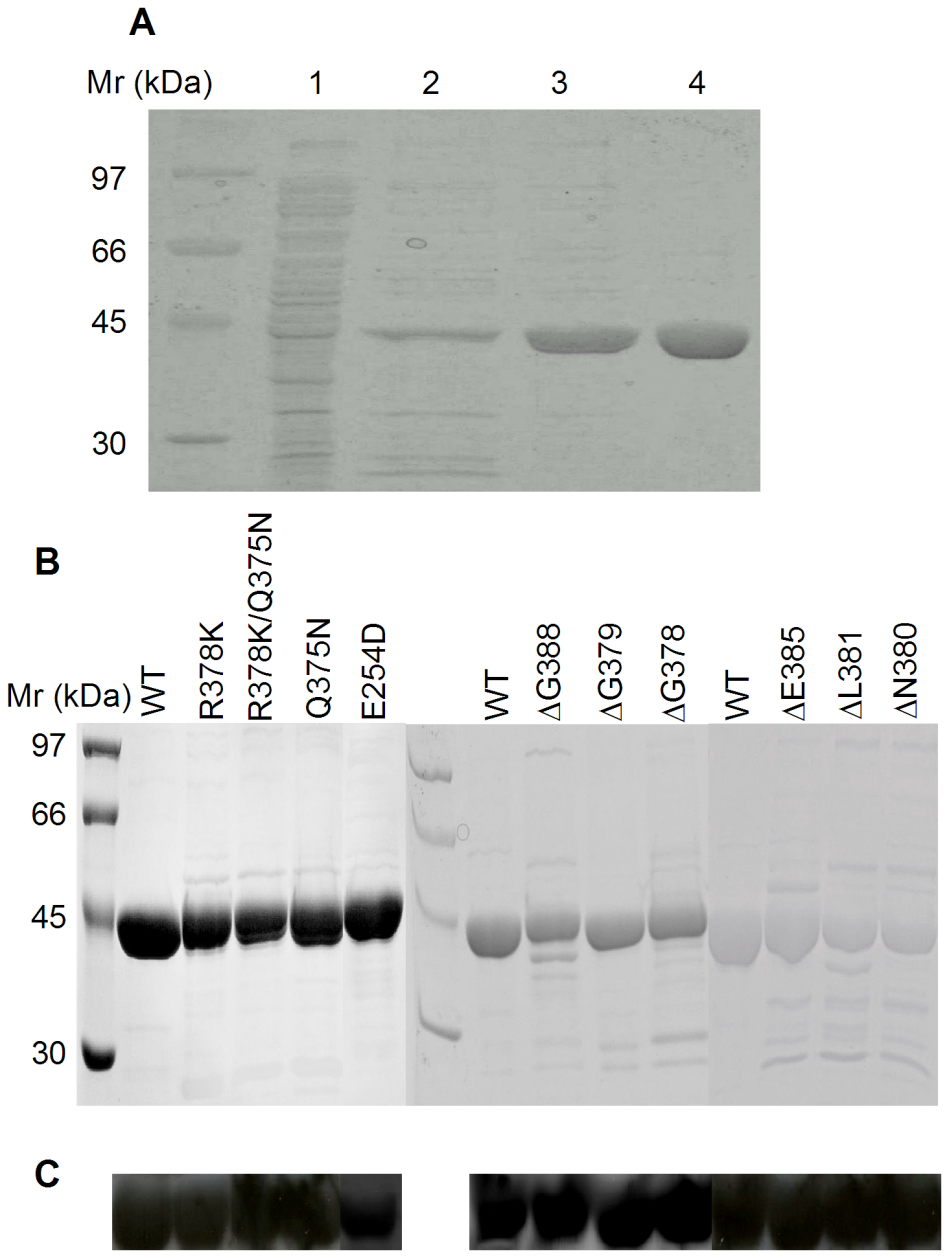
SDS-PAGE analyses of wild-type and mutant 4-HPPD enzymes. (A) Fractions are shown for the different purification steps of wild-type 4-HPPD. Lane 1, cell crude lysate; Lane 2, following Q-sepharose anion-exchange chromatography; Lane 3, following SOURCE 15PHE hydrophobic interaction chromatography; Lane 4, concentrated fractions after S-100 Sephacryl gel filtration chromatography. (B) Purified wild-type and mutant 4-HPPD enzymes. (C) Western blotting of (B).

Purification of recombinant 4-HPPD using anion-exchange, hydrophobic interaction and size-exclusion chromatography yielded a pure protein, as judged by SDS-PAGE analyses (Fig. 3A). The purified protein showed a single major band with molecular mass about 45 kDa on SDS-PAGE (Fig. 3B). The yield of purified 4-HPPD was about 10% based on enzyme activity, with about a 15-fold improvement in purity from the cell lysate (Table 2). The five residues of the N-terminus in recombinant 4-HPPD were determined to be TTYSD, consistent with that predicted from the gene sequence of the human liver enzyme [27]. Purified recombinant wild-type and mutant 4-HPPD enzymes were further confirmed to be the same enzyme by Western blotting analysis (Fig. 3C).

**Table 2:** The purification of recombinant human 4-HPPD.

Purification steps	Total activity (U)	Protein concentration (mg/mL)	Specific activity (U/mg)	Yield (%)	Purification factor
Crude extract	113	19	0.2	100	1
Step I^a^	53	1.0	1.2	47	7
Step II	18	0.9	2.4	16	14
Step III	12	0.9	2.5	10	15

a Steps I to III indicate the pooled fractions after the Q-Sepharose column, after the SOURCE 15PHE column and concentrated fractions pooled after the S-100 Sephacryl column, respectively. The 4-HPPD activity was measured by the formation of HG in µmol/min (U) using the HPLC assay.

### Characterisation of recombinant human 4-HPPD

The catalytic activity of purified recombinant 4-HPPD was determined by a HPLC assay, which measures the formation of homogentisate, and the Oxygraph assay which continuously measures oxygen consumption during catalysis. The requirement for a reducing agent in the assay was determined using the HPLC assay. The presence of ascorbate in stoichiometric amounts resulted in an increase in specific activity of the recombinant enzyme by *ca.* 2-fold. When the stoichiometry was increased to 4-fold, activity was increased by *ca.* 2.7-fold compared to in the absence of ascorbate. In contrast, addition of dithiothreitol (DTT) to the assay solution had no significant effect on activity. Addition of tris(2-carboxyethyl)phosphine (TCEP) reduced the activity by *ca.* 30%.

To determine the efficiency of recombinant 4-HPPD to convert 4-HPP substrate to HG product, production of 4-hydroxyphenylpyruvate (4-HPA) was determined. No 4-HPA was produced by the wild-type enzyme, indicating that this alternative product was not produced at a significant level. The specific activity of the wild-type enzyme was determined to be 2.6±0.1 and 2.8±0.1 µmol/min/mg by Oxygraph and HPLC assays, respectively (Table 3). These results are in agreement with the data reported for native human 4-HPPD [9], [27].

**Table 3:** Activities of the wild-type and mutant enzymes.

	Oxygen consumption assay		HG product assay	
proteins	Specific activity (µmol min^−1^ mg^−1^)	Relative activity (%)	Specific activity (µmol min^−1^ mg^−1^)	Relative activity (%)
Wild-type	2.6±0.1	100	2.8±0.1	100
ΔG388	1.5±0.2	56	2.2±0.1	81
ΔE385	1.7±0.1	65	1.6±0.1	57
ΔL381	1.1±0.3	42	1.4±0.1	50
ΔN380	0.8±0.03	31	0.5±0.02	17
ΔG379	0.1±0.02	5	0.03±0.01	1
ΔR378	ND	<0.1	ND	<0.1
R378K	0.07±0.01	3	0.04±0.01	1
Q375N	ND	<0.1	ND	<0.1
R378K/Q375N	ND	<0.1	ND	<0.1
E254D	0.06±0.01	2	0.2±0.02	7

The data are the mean ± S.D. of three independent experiments. ND, not detectable.

### Activity of mutant enzymes

To investigate the influence of the C-terminus on the catalytic function of 4-HPPD, several mutants were constructed with different numbers of residues removed: 5 residues (ΔG388); 8 residues (ΔE385); 12 residues (ΔL381); 13 residues (ΔN380); 14 residues (ΔG379); and 15 residues (ΔR378). The R378K, Q375N and R378K/Q375N point mutants were also constructed. All of these proteins were purified to near homogeneity, as judged by SDS-PAGE analyses (Fig. 3B). Circular dichroism analyses of all mutants suggested no gross changes in the secondary structure as compared to the wild-type enzyme.

Deletion of residues from the C-terminus resulted in a reduction in enzymatic activity (Table 3). The activities of the ΔG388, ΔE385 and ΔL381 mutants were only *ca.* 50 to 60% of that of the wild-type enzyme. The ΔN380 mutant was *ca.* 20% as active as the wild-type enzyme, and further truncation to R378 abolished all activity. The R378K or E254D mutation reduced activity to *ca.* 5% of the wild-type enzyme, and all activity was abolished in the Q375N and R378K/Q375N mutants, indicating the importance of these residues for catalysis. Production of HPA by these mutants was not detected, showing that the reduction in activity was not due to the formation of an alternative product.

Steady-state kinetic analyses on these mutants were carried out using the Oxygraph assay (Table 4). The *k*_cat_ and *K*_m_ values for 4-HPP substrate for wild-type enzyme was determined to be 2.2±0.1 s^−1^ and 0.08±0.02 mM, respectively. No significant change in *K*_m_ value was observed for the ΔG388, ΔE385 or ΔL381 mutants compared to wild-type enzyme, whilst *k*_cat_ values were reduced by *ca.* 25 to 50%. However, deletion of further residues (ΔN380 and ΔG379 mutants) resulted in increased *K*_m_ and decreased *k*_cat_ values. The latter two mutants had a catalytic efficiency of *ca.* 3% and 1% compared to wild-type enzyme, respectively, as judged by *k*_cat_/*K*_m_ values. Substitution in R378 or E254 resulted in about 95% reduction in *k*_cat_ values. In addition, the R378K mutant showed an increased *K*_m_ value.

**Table 4:** Apparent kinetic parameters of wild-type and mutant 4-HPPD enzymes measured using the oxygraph assay.

Proteins	*k*_cat_ (s^−1^)	*K*_m_ (mM)	*k*_cat_ */K*_m_ (s^−1^mM^−1^)
Wild-type	2.2±0.1	0.08±0.02	29±4
ΔG388	1.4±0.2	0.08±0.01	17±2
ΔL385	1.4±0.1	0.09±0.01	16±1.2
ΔL381	1.0±0.2	0.07±0.01	14±3.2
ΔN380	0.5±0.02	0.3±0.1	1.5±0.2
ΔG379	0.1±0.01	0.12±0.01	0.9±0.1
R378K	0.07±0.01	0.24±0.01	0.27±0.01
E254D	0.06±0.01	0.11±0.02	0.57±0.12

Reported data are mean ± S.D. of three independent experiments.

### Structural modeling

**Figure 4:**
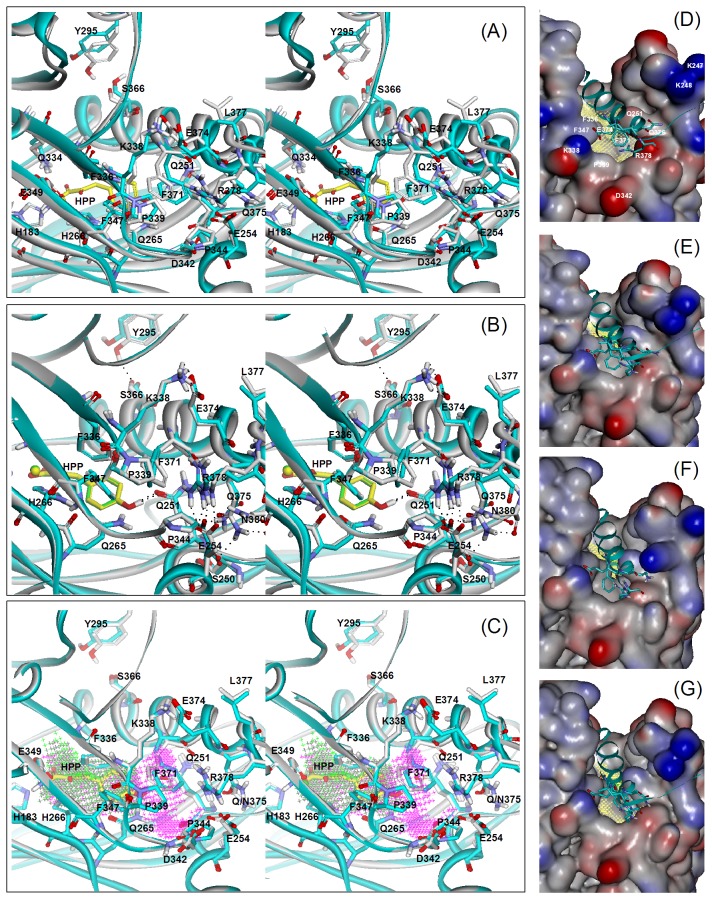
Models of 4-HPPD in complex with 4-HPP substrate. Superimposition of the model for the wild-type enzyme and the X-ray crystal structure of human 4-HPPD (PDB code: 3ISQ) [16] (A), and the models of ΔG379 (B), and Q375N (C) mutant enzymes (stereo image). The protein is shown as a cartoon and colored grey and cyan for the wild-type model and crystal structure or mutant models, respectively. The metal and 4-HPP present as sphere and stick models and colored green and yellow for wild-type and mutant enzymes, respectively. Hydrogen bonding interactions are shown as dashed black lines in (B). The putative substrate binding cavity is shown as Jacks style and colored green and magenta for wild-type and Q375N mutant models, respectively in (C). (D–G) Presentation of the crystal structure (D), wild-type (E), ΔG379 (F), and Q375N (G) models as surface styles and colored by interpolated charge. The C-terminus and residues in the final helix are shown as a ribbon and stick model, respectively, and colored in cyan. The cavity for putative substrate binding cavity is colored yellow. The metal and 4-HPP present as sphere and stick models and colored green.

The structure of human 4-HPPD was previously determined without bound substrate (Fig. 4A) [16]. Binding of the 4-HPP substrate into the active site of the enzyme was therefore modeled using a docking routine. The geometry minimization by QM-MM calculations was performed for all enzymes in complex with 4-HPP. Compared to the structure of 4-HPPD, this model with bound 4-HPP was shifted with the C_α_ r.m.s.d about 0.83 Å. The model of the enzyme in complex with substrate was in agreement with the previously proposed binding mode of 4-HPP [12], [25]. The oxygen atoms of the carboxyl and α-keto group of 4-HPP forms a bidentate interaction with iron at distances of 2.0 and 2.03 Å, respectively. The substrate aromatic side-chain is sandwiched between the phenyl rings of F336 and F364, and the phenolic 4-hydroxyl group of 4-HPP forms hydrogen bonds with the side-chains of Q265 and Q251 (Fig. 4B).

In the modeled structure of mutant protein, the structure the terminal α-helix was apparently shifted with core r.m.s.d. about 0.46, 0.45 and 0.28 Å for the ΔG379, Q375N and R378K mutants, respectively, as compared to the wild-type model. The side-chains of Q251, E254, Q265, Y295, F336, F364, F368, F371, Q375, L377 and R378 in ΔG379 mutant model were obviously moved with r.m.s.d. about 0.32, 1.1, 0.23, 0.54, 0.22, 0.25, 0.37, 0.86, 1.35, 1.39 and 0.78 Å, respectively (Fig. 4B). It is noted that the aromatic ring of F371 in this mutant model was particularly rotated by about 60 degrees. Due to the truncation, the bifurcate interactions between the side-chain of Q375 and the oxygen atom of S250 and N380 were disrupted and in turn new hydrogen bonds were formed between the side-chains of Q375 and E254. Similarly, Q375 formed new interactions with Q251 and N380 in the ΔN380 mutant model (data not shown). In the modeled structure of Q375N mutant, similar movement in the side-chains of these residues were shown. Moreover, the dramatic movement in the side-chains of F347 and F371 (about 0.47 and 2.16 Å, respectively) resulted in increased accessibility of the active site cavity. A channel extending from the active site to the C-terminus was shown in the modeled structure (Fig. 4C).

## Discussion

Human recombinant 4-HPPD was cloned from HepG2 cells and expressed as soluble protein in *E. coli*. The purified protein had similar enzymatic activity as the native enzyme. The recombinant enzyme was only activated by ascorbate, and not by other reducing agents. This contrasts with the results for native human liver 4-HPPD, where activation was observed for ascorbate and other reducing cofactors [9], [27]. Activation by ascorbate and DTT has been previously reported for rat liver α-ketoisocaproate oxidase [27], [30]. This α-ketoisocaproate oxidase was later found to be the same enzyme as 4-HPPD [27], [31]. Activation of enzyme activity by ascorbate has also been reported for other oxygenase enzymes, *e.g.* deacetoxycephalosporin synthase (DAOCS) and prolyl 4-hydroxylase [27], [32]–[34]. In these enzymes, ascorbate is probably used to keep the iron in the ferrous state in the active site. It has also been proposed that ascorbate is able to reduce the ferryl species during uncoupled catalytic cycles performed by prolyl-4-hydroxylase and 2,4-dichlorophenoxyacetic acid/2-oxoglutarate dioxygenase [27], [33], [34].

The active site of 4-HPPD is enclosed by a C-terminal α-helix which is assumed to function as a gate which controls access of substrate [11]. The C-terminus of the reported structure was resolved only as far as Met-384 and the last 9 residues were disordered and not visible in the structure (Fig. 4A) [16]. Arg-378 is located at the end of the final α-helix, and the last 15 residues comprise the disordered C-terminal tail. Previously Lee *et. al.* [26] identified a mutant rat 4-HPPD gene that encoded a protein with 14 residues deleted from the C-terminus, which was expressed as an inactive enzyme. This mutant showed no brownish pigment formation when expressed in *E. coli* and had no detectable decarboxylation ability using α-ketoisocaproate as a substrate. This study shows that all activity is lost for human 4-HPPD upon removal of the final 15 residues (ΔR378) of the C-terminus when 4-HPP was used as substrate. Human and rat 4-HPPD have the same number of amino acids in their sequences and are *ca.* 85% identical. The results in this study show the critical role of this disordered tail for enzyme activity in these enzymes. This study also shows that the last 5 residues, VVPGM, of the C-terminus are important for enzyme activity. Deletion of these residues led to loss of *ca.* 50% activity. These mutants had very similar *K*_m_ values to the wild-type enzyme but *k*_cat_ values were significantly reduced. Even more reduction of *k*_cat_ values were observed for the ΔL381 and ΔN380 and ΔG379 mutants. The results suggest that the interactions provided by the last five residues, L381 and N380 are critical for the catalysis.

Inspection of the reported structure of human 4-HPPD determined without bound substrate shows that the putative substrate binding site is broad and partly formed by the C-terminus (Fig. 4D). Docking of substrate into the putative binding site appears to induce a conformational change. The simulation model shows that the orientation of the amide group in the side-chain of Q251 is reversed allowing interaction with substrate. In this manner the side-chain of Q251 is located near the aromatic ring of F336 and both residues act as a gate to restrict access to the substrate binding pocket (Fig. 4A and E). This change seems drive the movement of the final helix (about 2 Å) with formation of a new salt bridge between the side-chains of R338 and E374 and a new hydrogen bond between the side-chains of S366 and Y295. Residues of F347, F371, P339, R378 and Q375 that line the channel from the gate to the C-terminus were shifted about 0.7, 2.1, 1.2, 1.5 and 1.5 Å, respectively, to shield the active site from solvent. It is noted that the shift of Q375 led to the switch of its bifurcate interactions with N380 and K249 to N380 and S250 after substrate binding. The tail in the C-terminus is assumed to interact with residues surrounding the active site entrance but it is not visible in this model.

In the structure of human 4-HPPD, residues of N380, L381, T382 and N383 in the tail participate in interactions with residues of E254, Y258, Q375, K247, K248 and K249, which are located around the entrance of active site, to fix the end of the final helix into the appropriate position (Fig. 4A) [16]. The ΔG379 model showed the truncation of the tail to disrupt these interactions, causing apparent movements of the final α-helix and residues near the active site entrance. In particular a conformational change in the aromatic ring of F371 and large differences in the conformations of E254, R378 and Q375 are predicted (Fig. 4B). The distance between the side-chain of Q251 and F336 was increased about 0.3 Å as compared with wild-type model. The broad access might be covered by residues lining the channel. However, due to the change in conformation in the aromatic ring of F371, the positions of F347 and P339 were subtle moved (Fig. 4F). Truncation of the tail might expose the entrance and increases the solvent accessibility to the active site. This change might be the reason for loss of catalytic activity. Results from kinetics of tail truncated enzymes suggest the interactions provided by the last residues together with L381 and N380 are critical for catalysis. The structural models suggest residues of Q375, K248 and D342 located around the active site entrance might interact with these residues in the tail. Furthermore, the crystal structure and models of wild-type or the different truncated mutants showed that Q375 can have various interactions with the tail and residues near the active site entrance, suggesting the dynamic interaction provide by this residue might mediate the position of final helix and C-terminus during the catalytic cycle.

Residues in the terminal α-helix of 4-HPPD may also have a role in catalysis. A previous study by Gunsior *et al* [23] showed that *ca.* 98 and 80% of activity was lost for the *sa*4-HPPD F337I and F341Y mutants, respectively. The F337I mutant also catalyzed the formation of oxepinone (from an arene oxide-derived intermediate) and hydroxymandelate (HMA) in addition to HG. These two residues correspond to F364 and F368 in the terminal helix of *h*4-HPPD. The results in this study indicate that the π-π interaction between the 4-HPP substrate and F364 is important for maintaining the benzene ring of 4-HPP in a proper position for oxidation [23], [25]. In the present study, the activity lost by substitution of R378 and Q375 indicates a strict requirement of these two residues in the proper position for catalysis. These two residues are not conservative among different species and their equivalent residues in plants are Leu and Glu, respectively (Fig. 2). In the *h*4-HPPD structure, R378 and Q375 are located close to the C-terminal end of the helix facing the entrance of the active site, and Q375 is situated near the Q251 (about 3.5 Å) [16]. Q251 is a critical catalytic residue which forms hydrogen bonds with the 4-hydroxy group of the 4-HPP substrate. Studies by Raspail *et al.* [25] showed that the interactions provided by Q272, Q286 and Q358 (corresponding to Q251, Q265 and Q334 in human 4-HPPD) are critical for the formation of the enzyme-4-HPP complex and the first nucleophilic reaction by dioxygen.

In the wild-type model, Q375 forms bifurcate hydrogen bonds with S250 and N380 to fix the final helix and the tail in the appropriate position, substitution to disrupt these interactions would destabilize the C-terminus. In the Q375N mutant model, the side-chain of Q251 and F336 was separated for about 0.4 Å wider than that in the wild-type model and the aromatic ring of F371 was dramatic shifted away from that of F347 (Fig. 4E and G). These changes resulted in opening a solvent accessible channel from the putative substrate binding pocket to the C-terminus. Furthermore, without these interactions to stabilize the tail, it might not function properly to cover the active site entrance. This may account for the complete loss of activity.

R378 is the final residue in the terminal helix. In the structure, this residue interacts with E254 to fix the terminal helix in proper position (Fig. 4A). Mutation of R378 or E254 resulted in most of the activity lost especially the reduction of the *k*_cat_ value. The R378K mutant model showed that the substitution not only disrupted the interaction between R378 and E254 but also affected the interaction between Q375 and S250 and the hydrogen bonding of Y259 and S366. These results indicating the interactions formed by the two residues are critical for maintaining the terminal helix in a stabile conformation for catalysis. However, inspection the models of truncated and Q375N mutant enzymes, show that although the relative positions of R378 and E254 are shifted, the interactions between the two residues are still retained. The result highlights the roles of R378 and Q375 in stabilization of the C-terminus are different.

The models and kinetic data suggest the bifurcate interactions provided by Q375 are critical to stabilize the final α-helix and the tail in the correct position for catalysis. Hence, truncation of the C-terminus means that the final α-helix cannot appropriately function as a gate to isolate the active site from solvent and hence impacts on the catalytic reaction performed by mutant 4-HPPD enzymes. Mutant enzymes which are still active produce the expected HG product, suggesting that the orientation of the substrate aromatic ring is still appropriate. Removal of part or all of the C-terminal tail might affect either step in the catalytic cycle including the activation of dioxygen, the nucleophilic attack by dioxygen or the release of the HG product; the latter step has been shown to be rate-limiting for the reaction [35]. However, this hypothesis requires further confirmation. Results from this study suggest that other roles for the C-terminus are also possible. Through the interaction with Q375, the dynamic orientation of the tail might mediate the appropriate position of the final α-helix to ensure that gate is opened during the appropriate steps in the catalytic cycle to allow substrate to bind and the release of product [11], [36]. These interactions assume to maintain the integrity of the active site, ensuring correct orientation of substrate and its interaction with residues in the active site which are important for catalysis.
